# An X chromosome-wide association study in autism families identifies *TBL1X *as a novel autism spectrum disorder candidate gene in males

**DOI:** 10.1186/2040-2392-2-18

**Published:** 2011-11-04

**Authors:** Ren-Hua Chung, Deqiong Ma, Kai Wang, Dale J Hedges, James M Jaworski, John R Gilbert, Michael L Cuccaro, Harry H Wright, Ruth K Abramson, Ioanna Konidari, Patrice L Whitehead, Gerard D Schellenberg, Hakon Hakonarson, Jonathan L Haines, Margaret A Pericak-Vance, Eden R Martin

**Affiliations:** 1 Hussman Institute for Human Genomics, Miller School of Medicine, University of Miami, PO Box 019132 (M-860), Miami, FL 33101, USA; 2 Center for Applied Genomics, Children's Hospital of Philadelphia, 34th St and Civic Center Blvd, Philadelphia, PA, 19104, USA; 3 School of Medicine, University of South Carolina, 6311 Garners Ferry Road, Columbia, SC, 29209, USA; 4 Department of Pathology and Laboratory Medicine, University of Pennsylvania, 3620 Hamilton Walk, Philadelphia, PA, USA; 5 Center for Human Genetics Research, Vanderbilt University Medical Center, 2215 Garland Ave, Nashville, TN, 37232, USA

## Abstract

**Background:**

Autism spectrum disorder (ASD) is a complex neurodevelopmental disorder with a strong genetic component. The skewed prevalence toward males and evidence suggestive of linkage to the X chromosome in some studies suggest the presence of X-linked susceptibility genes in people with ASD.

**Methods:**

We analyzed genome-wide association study (GWAS) data on the X chromosome in three independent autism GWAS data sets: two family data sets and one case-control data set. We performed meta- and joint analyses on the combined family and case-control data sets. In addition to the meta- and joint analyses, we performed replication analysis by using the two family data sets as a discovery data set and the case-control data set as a validation data set.

**Results:**

One SNP, rs17321050, in the transducin β-like 1X-linked (*TBL1X*) gene [OMIM:300196] showed chromosome-wide significance in the meta-analysis (*P *value = 4.86 × 10^-6^) and joint analysis (*P *value = 4.53 × 10^-6^) in males. The SNP was also close to the replication threshold of 0.0025 in the discovery data set (*P *= 5.89 × 10^-3^) and passed the replication threshold in the validation data set (*P *= 2.56 × 10^-4^). Two other SNPs in the same gene in linkage disequilibrium with rs17321050 also showed significance close to the chromosome-wide threshold in the meta-analysis.

**Conclusions:**

*TBL1X *is in the Wnt signaling pathway, which has previously been implicated as having a role in autism. Deletions in the Xp22.2 to Xp22.3 region containing *TBL1X *and surrounding genes are associated with several genetic syndromes that include intellectual disability and autistic features. Our results, based on meta-analysis, joint analysis and replication analysis, suggest that *TBL1X *may play a role in ASD risk.

## Background

Autism spectrum disorder (ASD) is a complex disorder of neurodevelopmental origin which is characterized by a well-established set of social, communicative and behavioral impairments [1]. These impairments confer a significant burden on individuals with ASD and their families. This burden, in conjunction with a high ASD prevalence (about 1 in 100 children ages 3 to 17 years in the United States) [2], has spurred aggressive efforts to identify ASD risk genes. Often reported but poorly understood clinical phenomena with implications for gene discovery efforts in ASD is the high male:female ratio. Although this finding strongly suggests an etiological role for the sex chromosomes [3,4], there has been limited success in understanding the role of the sex chromosomes associated with ASD risk.

Genome-wide linkage studies have implicated regions on the X chromosome [5,6], and identification of structural variants in genes such as neuroligin 4, X-linked (*NLGN4X*) demonstrate the potential role of X-linked genes in autism [7]. Despite these results, gene-mapping studies have focused on the autosomes, perhaps owing in part to the analytical difficulty of studying the X chromosome in complex diseases. Taking advantage of statistical methods specifically developed for this challenge [8], we investigated the X chromosome on a chromosome-wide level in three recently completed genome-wide association study (GWAS) data sets [9,10].

Herein we report our analysis of chromosome-wide data on the X chromosome for association with ASD in two independent family-based samples as well as in a third independent sample of unrelated cases and controls. We conducted meta-analysis and a novel approach to joint analysis in the combined family and case-control samples. We also combined the two family samples as a discovery data set and used the case-control samples as a validation data set and performed comprehensive analysis in the discovery and validation data sets to establish replication. Furthermore, we investigated whether there are SNPs in previously reported candidate genes for ASD that show replication among the discovery, validation and joint data sets.

## Subjects and methods

### Study samples

Three data sets were used in this study. A total of 894 ASD families (3,128 individuals) ascertained by three clinical groups at the John P. Hussman Institute for Human Genomics (HIHG; Miami, FL, USA), the University of South Carolina (Columbia, SC, USA) and the Vanderbilt Center for Human Genetics Research (CHGR; Vanderbilt University, Nashville, TN, USA), denoted collectively as HIHG/CHGR, were included in this study. We combined this data set with a second GWAS data set obtained from the Autism Genetic Resource Exchange (AGRE). The full AGRE data set is publicly available and comprises families with ASD. There were 939 ASD families (4,495 individuals) included in the AGRE GWAS. The third data set was from the Autism Case-Control (ACC) cohort study conducted at the Children's Hospital of Philadelphia (CHOP) [9]. The ACC cohort data were collected from multiple sites across the United States, including CHOP. The ACC controls had no history of ASD and were of self-reported Caucasian ancestry. The data set consisted of a total of 1,204 cases and 6,472 controls. Detailed diagnostic criteria for the HIHG/CHGR data set can be found in the study by Ma *et al*. [10], those for the AGRE data set can be found on the AGRE website (http://www.agre.org/) and those for the ACC data set can be found in the study by Wang *et al*. [9]. In brief, the core inclusion criteria for the HIHG/CHGR data set included (1) chronological age between 3 and 21 years of age, (2) a presumptive clinical diagnosis of ASD and (3) expert clinical determination of an ASD diagnosis based on *Diagnostic and Statistical Manual of Mental Disorders, Fourth Edition, Text Revision *(DSM-IV-TR) [1] criteria and supported by the Autism Diagnostic Interview-Revised (ADI-R) in the majority of cases and all available clinical information. For AGRE, families with one or more individuals diagnosed with ASD (based on DSM-IV-TR and ADI-R) were selected. For ACC, individuals with positive ADI/ADI-R or ADOS, or both, were included. Detailed statistics for subphenotypes such as autism, ASD and mean IQ for the data sets can be found in Additional file 1.

### Molecular methods

The HIHG/CHGR data set was genotyped in the genotyping core at the Center for Genome Technology at HIHG. Samples passing sample quality checks were genotyped using the Infinium Human1M version 1 BeadChip Kit (Illumina Inc, San Diego, CA, USA), which contains 1,072,820 SNPs (of which 36,377 loci are on the X chromosome). The samples were processed in batches of 48 according to Illumina procedures for processing of the Infinium II assay. The above protocol was automated using the Freedom EVO robotic system (Tecan Group Ltd, Männedorf, Switzerland) to further enhance the efficiency and consistency of the assay. A common quality control (QC) DNA sample was repeated during each run to ensure the reproducibility of results between runs. Data were extracted by using Illumina BeadStudio software from data files created by the Illumina BeadArray Reader. Samples and SNPs with call rates below 95% were excluded from analysis, and an Illumina GenCall Data Analysis software cutoff score of 0.15 was used for all Infinium II products (Illumina Inc) [10]. The AGRE and ACC samples were genotyped using the Illumina HumanHap550 Genotyping BeadChip SNP genotyping array, which has 13,837 X-chromosome SNPs from among a total of 555,175 SNPs. A more detailed description of genotyping procedures used to analyze the AGRE and ACC samples can be found in the study by Wang *et al*. [9].

### Sample quality control

In the HIHG/CHGR and AGRE data sets, individuals' sex was determined on the basis of the X-chromosome SNPs. Genome-wide identity by descent estimates were used to test for relatedness between samples to detect other sample errors. Families with a genome-wide Mendelian error rate greater than 2% across SNPs were also excluded from the analysis. These procedures were performed using the PLINK software [11].

EIGENSTRAT [12] was used to identify population stratification and remove outliers. We performed EIGENSTRAT on autosomes of the parents in the HIHG/CHGR and AGRE data sets. Families that had individuals with eigenvectors in the first four principal components beyond four standard deviations of the mean were considered outliers and were removed from the data. Only families of Caucasian descent (based on self-report) passed the outlier standard. Families from other racial groups, such as African-Americans and Asian-Americans were removed as outliers. Similar procedures for sample QC were performed on the ACC data set from CHOP [9].

### SNP quality control

We used PLINK software to remove from the HIHG/CHGR and AGRE data sets SNPs with Mendelian error rates greater than 4% and *P *values less than 10^-4 ^for the Hardy-Weinberg Equilibrium tests. SNPs with minor allele frequency less than 0.01 were also excluded, since the power to detect association with such rare variants was low in this sample.

We identified several SNPs with significantly different rates of missing genotypes in males and females where the missing data are non-random with respect to genotype. Since the prevalence of ASD is higher in males than in females, the difference in missing genotype rates between males and females and genotype-specific missingness can change allele frequencies between individuals with ASD versus those without ASD, which can lead to spurious association results. Therefore, to reduce the effect of informative missingness, we excluded markers with a missing genotype rate more than 2.5% in males or females.

In general, males are hemizygous for the X chromosome, except for three homologous regions shared by the X and Y chromosomes. Markers in the two telomeric pseudoautosomal regions (PAR1 and PAR2) were treated as autosomal markers in the QC and analysis. A third region of XY homology, Xq21.3 and Yp11.1, as well as several other smaller segments [13], differs from the PARs in that the X and Y chromosomes do not recombine. This can result in different allele frequencies in these regions on the X and Y chromosomes, which can lead to spurious results in association tests. Therefore, SNPs in this region and those showing more than 1% male heterozygotes were removed from our analysis. Male heterozygotes in the remaining SNPs may have been due to errors in genotype calls, thus their genotypes at that SNP were set to be missing.

### Statistical analysis

For joint analysis of the pooled data sets (HIHG/CHGR, AGRE and ACC), we used a modified version of X-APL [8] that combines family and unrelated case-control data [14]. Briefly, unrelated cases and controls were integrated into the family-based framework by treating each as a proband from a family triad with missing parental data. In this way, they contributed indirectly to inferences regarding missing parental data across the sample and directly to inferences about ASD-SNP associations. We found that the joint analysis resulted in a high inflation factor (λ > 1.6) based on the genomic control (GC) approach [15]. The high inflation factor could be due to the batch effect in the ACC data set, as discussed below, but it also could be due to the fact that QC analyses of population stratification for family and case-control data were performed separately at two sites (HIHG/CHGR and CHOP). Applying the GC approach to the joint analysis results had a drastic effect on the power to detect true positives. Therefore, we performed stratified analysis by grouping HIHG/CHGR and AGRE into one cluster and ACC into the other cluster in the joint analysis. Allele frequencies were inferred individually in the two clusters, and an X-APL statistic was calculated for each cluster. The statistics from the two clusters were summed to form a final statistic, and variance for the statistic was estimated jointly on the basis of the data derived from the two clusters. We also used a meta-analysis approach comprising steps similar to those described by Wang *et al*. [9] to combine the X-APL statistics from the HIHG/CHGR and AGRE cohorts with the GC-corrected statistics from the ACC sample.

For the replication analysis, association tests in the individual data sets were conducted using the PLINK software [11] for case-control analysis based on allele-based χ^2 ^tests and X-APL in families [8]. APL [16] and allele-based χ^2 ^tests for autosomes using the PLINK software were performed to detect SNPs in PAR1 and PAR2. Unaffected siblings were also included in family-based analyses, as they helped us infer missing parental genotypes. The analysis of the ACC data set had an inflation factor (that is, λ value [15]) of 1.12, which may reflect a batch effect, since cases and controls were genotyped in separate batches as described in the supplement section of the study by Wang *et al*. [9]. We applied the GC approach to the results for the case-control analysis as suggested by Wang *et al*. [9] to correct for the batch effect. We used the combined family data set (HIHG/CHGR and AGRE) as our discovery data set and the case-control data set (ACC) as the validation data set. We performed individual analyses of the discovery and validation data sets and investigated whether the results were replicated in both data sets.

We also investigated whether there were significant signals in candidate genes identified in previous studies for interesting, albeit not chromosome-wide, significant SNPs. SNPs with *P *values less than 0.05 in the discovery, validation and joint analyses as well as in candidate genes found in the literature were considered significant. A total of 21 candidate genes for ASD on the X chromosome reported in the Autism Genetic Database [17] were considered. A list of the 21 candidate genes are given in Additional file 2. On the basis of considering only 776 SNPs in the 21 candidate genes from among the 10,820 overlapping SNPs in the discovery, validation and joint data sets, we expected that the chance of a SNP being significant in the discovery, validation and joint data sets and in a candidate gene would be small. A diagram depicting the analytical strategy is shown in Additional file 3.

To investigate whether there are sex-specific effects in ASD, we also performed case-control tests for males and females separately. For family-based analysis, we performed sex-specific tests proposed by Chung *et al*. [8] by calculating the transmission of alleles from parents to affected males and affected females separately. Because cases and controls were integrated into the family-based framework, male- and female-specific tests were calculated similarly in the joint analysis based on the modified version of X-APL.

Current imputation methods have been developed mainly for unrelated samples, which were not suitable for analysis of the HIHG/CHGR and AGRE data sets. Recently, the genotype imputation method BEAGLE [18] was proposed to study triads and unrelated individuals; however, this method is restricted to autosomal SNPs and family triads. Given these practical restrictions and the fact that there was still good coverage with the overlapping SNPs (10,820 SNPs), we analyzed only SNPs common to the three data sets for the meta- and joint analyses.

The use of a traditional Bonferroni correction for multiple testing was conservative because of linkage disequilibrium (LD) among markers. We used the simpleM method based on principal component analysis to choose the "effective number" of independent tests [19]. The *P *values corrected using the simpleM method were similar to those corrected by the permutation procedure for multiple testing [19]. The effective number for the HIHG/CHGR data set was about 11,500 and that for the AGRE, ACC and joint data sets was about 8,000. With the chromosome-wide correction set at 0.05 based on the simpleM method, the SNP-wise significance levels were approximately 4.3 × 10^-6 ^for the HIHG/CHGR analysis and approximately 6.25 × 10^-6 ^for the AGRE, ACC and joint analysis. For replication analyses, since the discovery data set (HIHG/CHGR and AGRE) and validation data set (ACC) were independent, we considered a replication threshold of $\sqrt{6.25\times 10^{-6}}=0.0025$. The results were replicated if their *P *values were less than 0.0025 in both the discovery and validation data sets. This assures a chromosome-wide type I error rate of no more than 0.05 for replicated results. Table 1 summarizes the significance thresholds for the meta-analysis, joint analysis, replication analysis and candidate gene analysis.

**Table 1:** Thresholds for significance in meta- and joint analyses, replication analysis and candidate gene analysis

Analysis type	Threshold for significance
Meta- or joint analysis	*P *< 6.25 × 10^-6 ^in either the meta- or joint analysis
Replication analysis	*P *< 0.0025 in both discovery and validation analyses
Candidate gene analysis	*P *< 0.05 in discovery, validation and joint analyses, and the SNP is in a candidate gene for autism

## Results

### Quality control results

The final data sets included 2,557 samples from 735 ASD families in the HIHG/CHGR data set, 3,289 samples from 721 ASD families in the AGRE data set and 1,204 cases and 6,472 controls from the ACC data set. The HIHG/CHGR data set had 620 singleton families (parent-child trio) and 115 multiplex families (more than one affected sibling). The AGRE data set had 138 singleton families and 583 multiplex families. After applying the QC filters, the following data sets remained: 24,712 X-chromosome SNPs (including 356 SNPs in PAR1 and PAR2) remained in the HIHG/CHGR data set, with an average call rate of 99.75%; 11,164 X-chromosome SNPs (including 15 SNPs in PAR1 and PAR2) remained in the AGRE data set, with an average call rate of 99.62%; and 11,098 X-chromosome SNPs (including 15 SNPs in PAR1 and PAR2) remained in the ACC data set, with an average call rate of 99.61%. In the three data sets, there were 10,820 overlapping X-chromosome SNPs for the meta- and joint analyses. The ratios of affected males to affected females in the HIHG/CHGR, AGRE and ACC data sets were 4.97, 3.82 and 4.6, respectively. The ratio of male controls to female controls in the ACC data set was 1.1. The detailed QC results from different procedures for samples and genotypes are given in Additional file 4.

### Meta-analysis and joint analysis

**Figure 1:**
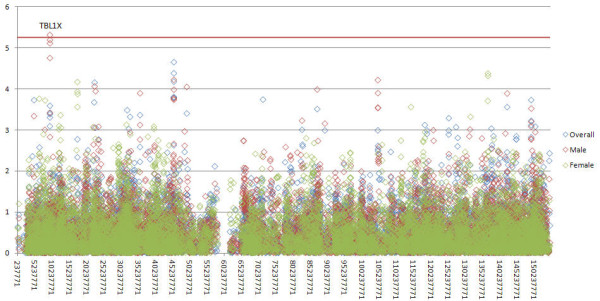
**Plot for -log_10 _(*P *values) for the overall, male-specific and female-specific tests based on the meta-analyses**. The red line indicates the threshold for chromosome-wide multiple testing correction.

**Figure 2:**
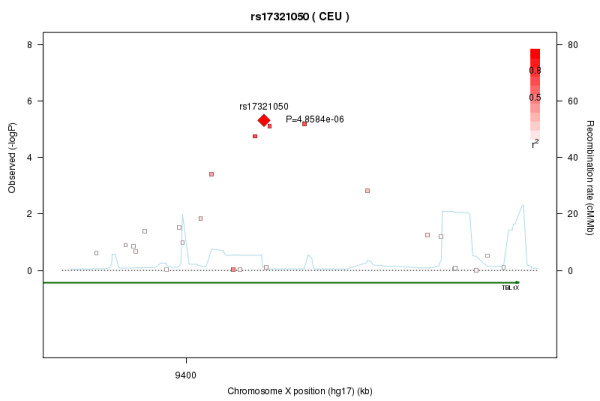
**Plot for the *P *values and LD structures for SNPs surrounding the three significant SNPs in *TBL1X***. The plot was generated using SNAP software [45]. LD = linkage disequilibrium; *TBL1X *= transducin β-like 1X-linked gene.

Figure 1 shows the plot of the *P *values for the overall, male-specific and female-specific tests based on the meta-analysis. The SNP rs17321050 in the transducin β-like 1X-linked (*TBL1X*) gene shows the chromosome-wide significance with a *P *value of 4.86 × 10^-6 ^for the male test. The joint analysis also identified the same SNP, rs17321050, with a chromosome-wide statistically significant *P *= 4.53 × 10^-6 ^for the male test. Notably, the *P *value for this SNP also was very close to the replication threshold in the discovery data set and passed the replication threshold in the validation data set, as shown in Table 2. We identified two other SNPs (rs5934665 and rs2188766) in the *TBL1X *gene that were close to the chromosome-wide significance in the meta-analysis for males (Table 2). These two SNPs were also close to the replication threshold of 0.0025 in both the discovery and validation data sets. These two SNPs, rs5934665 and rs2188766, showed evidence of LD in the joint data set with rs17321050 at *r*^2 ^= 0.64 and *r*^2 ^= 0.65, respectively (Additional file 5). Figure 2 shows the *P *values for males and the LD structures of the SNPs in the region. We show the allele and genotype frequencies for the three markers in parents, cases and unrelated controls in Additional file 6. We also performed a haplotype test for males for the three markers, but the association was not as significant as the single-marker tests (*P *= 0.001 for the global test in the meta-analysis). As shown in Table 2 the three SNPs were not significant for females.

**Table 2:** Results for the SNPs that survived chromosome-wide multiple testing in the meta- and joint analyses and for the two neighboring significant SNPs

Marker	Map position (bp)	Minor/major allele	MAF^a^	Discovery	Validation	Meta	Joint	Test	Gene
rs5934665	9,570,450	G/A	0.433	2.87 × 10^-3^	1.87 × 10^-3^	1.77 × 10^-5^	3.00 × 10^-5^	Male	*TBL1X*
rs5934665	9,570,450	G/A	0.449	2.46 × 10^-1^	3.24 × 10^-1^	8.59 × 10^-1^	4.67 × 10^-1^	Female	*TBL1X*
rs17321050	9,573,099	G/T	0.335	5.89 × 10^-3^	2.56 × 10^-4^	4.86 × 10^-6^	4.53 × 10^-6^	Male	*TBL1X*
rs17321050	9,573,099	G/T	0.359	1.99 × 10^-1^	5.22 × 10^-1^	6.15 × 10^-1^	3.85 × 10^-1^	Female	*TBL1X*
rs2188766	9,574,739	C/T	0.432	4.33 × 10^-3^	4.42 × 10^-4^	7.76 × 10^-6^	2.00 × 10^-5^	Male	*TBL1X*
rs2188766	9,574,739	C/T	0.451	2.34 × 10^-1^	3.80 × 10^-1^	7.86 × 10^-1^	4.21 × 10^-1^	Female	*TBL1X*

^a^Minor allele frequency (MAF) estimated on the basis of the discovery data set. *TBL1X *= transducin β-like 1X-linked gene.

### Replication analysis

Individually, no SNPs met the stringent threshold for multiple testing in either the discovery or validation data sets, nor did any SNPs pass the replication threshold in both the discovery and validation data sets.

### Candidate gene analysis

The results for significant SNPs in candidate genes are shown in Table 3. We identified SNP rs721699 in the Duchenne muscular dystrophy gene *Dystrophin *(*DMD*) [OMIM:300377] in the overall test and SNPs rs9887672, rs10218388 and rs5962575 in the IL-1 receptor accessory protein-like 2 (*IL1RAPL2*) gene [OMIM:300277] in the male test. The allele and genotype frequencies of the SNPs in parents, cases and unrelated controls are given in Additional file 6.

**Table 3:** Results for SNPs with replication and meta- and joint analysis *P *values < 0.05 and within candidate genes or regions

Marker	Map position (bp)	Minor/major allele	MAF^a^	Discovery	Validation	Meta	Joint	Test	Gene
rs721699	32,468,253	T/C	0.384	7.45 × 10^-3^	4.72 × 10^-2^	9.57 × 10^-4^	5.70 × 10^-4^	Overall	*DMD*
rs9887672	104,882,811	T/C	0.103	2.17 × 10^-2^	1.53 × 10^-3^	1.28 × 10^-4^	1.20 × 10^-3^	Male	*IL1RAPL2*
rs10218388	104,875,605	C/T	0.107	3.08 × 10^-2^	2.64 × 10^-3^	2.94 × 10^-4^	2.5 × 10^-4^	Male	*IL1RAPL2*
rs5962575	104,868,160	C/T	0.107	3.44 × 10^-2^	2.19 × 10^-3^	2.86 × 10^-4^	2.30 × 10^-4^	Male	*IL1RAPL2*

^a^Minor allele frequency (MAF) estimated on the basis of the discovery data set. *DMD *= *Dystrophin *Duchenne muscular dystrophy gene; *IL1RAPL2 *= IL-1 receptor accessory protein-like 2 gene.

## Discussion

Our study represents the largest chromosome-wide study of the X chromosome in association with ASD. Combining three independent GWAS studies allowed us to thoroughly evaluate the X chromosome using meta- and joint analyses in the pooled sample to improve our study's power to detect true-positives. Also, the replication and candidate gene analyses were used to further filter out false-positives. We found one intronic SNP, rs17321050, in *TBL1X *that demonstrated chromosome-wide evidence of association in the meta- and joint analyses, strongly supporting *TBL1X *as a risk factor for ASD. This finding was further supported by the replication analysis. Our estimates of the odds ratios for rs17321050 in *TBL1X *for males, based on the major allele as the reference, are 0.85 (95% confidence interval (95% CI) = 0.74 to 0.99) in the discovery data set and 0.74 (95% CI = 0.63 to 0.86) in the validation data set. Though modest, these effects were consistent across samples and similar to effect sizes seen for other significant regions in other recent GWASs of complex diseases [9,20]. One explanation for the modest odds ratios is that these associations reflect rare variants (structural variations or copy number variants (CNVs)) with very strong effects on ASD in LD with the significant SNPs. Follow-up sequencing might be warranted to identify the rare variants. Alternatively, the modest odds ratios might simply be due to the fact that the autism loci in LD with the SNPs have modest effects on the disorder. Moreover, the major allele in rs17321050, which is the positively associated allele, may be in LD with the risk alleles in the autism loci. Also, the SNP could have an unknown regulatory effect, as it is intronic with no predicted function. We performed a power study based on the sample sizes and show the power curves in Additional file 7. The power study suggests that, given the sample size and relative risk greater than 1.3, the test has more than 70% power to detect the true signal when the ASD allele frequency is greater than 0.3. The power study further demonstrates that the study design has the power to detect markers with modest effects on ASD.

The peak result in *TBL1X *is particularly interesting because *TBL1X *is in the Wnt signaling pathway [KEGG pathway ID:hsa04310]. The Wnt family of proteins is a highly conserved group of genes that are key mediators of cell-cell signaling during embryogenesis and play an essential role in the generation of normal embryos. Many WNT receptors are expressed during development and in the adult central nervous system. Previously published experiments have also suggested that the Wnt signaling pathway may function differently in brain regions based on expression analysis in mouse brain [21]. *TBL1X *and its family member transducin β-like 1 X-linked receptor 1 (*TBL1XR1*) [OMIM:608628] have been shown to interact with β-catenin and bind to the promoter of Wnt target genes induced by Wnt signaling [22]. *Engrailed 2 *(*EN2*) [OMIM:131310], a Wnt target gene [23], has been associated with autism in several studies [24-27]. The *WNT2 *gene (Wingless-type mouse mammary tumor virus integration site family, member 2) [OMIM:147870] is a candidate gene for autism [28]. Therefore, *TBL1X *plays a role in pathways that may be critical to the etiology of autism and is an excellent candidate gene.

The region containing *TBL1X *carries CNVs that have been associated with a diverse range of neurodevelopmental phenotypes. Multiple studies have shown that deletions in the Xp22.2 to Xp22.3 distal region that contain *NLGN4 *and *TBL1X *are associated with autism. Researchers in one study found deletions encompassing the Xp22.3 region in three autistic females [29]. Investigators in another study identified a 5.5-Mb deletion in the Xp22.2 to Xp22.3 region in a female who had autism, moderate mental retardation and some dysmorphic features [30]. A familial deletion in the Xp22.2 to Xp22.3 region has been found to be associated with a variable phenotype, including autism, in female carriers [31]. *TBL1X *is about 5 Mb from the *NLGN4 *gene [OMIM:300427]. Researchers in several studies have suggested that deletions or point mutations in *NLGN4 *are associated with autism [32,33]. Furthermore, *TBL1X *is partially or completely deleted in patients with the ocular albinism with late-onset sensorineural deafness (*OASD*) gene [OMIM:300650] carrying Xp22.3-terminal deletions. Given these findings, we used PennCNV [34] to identify possible CNVs in the Xp22.2 to Xp22.3 region in our data set. Only one affected female sibling carried a single-copy duplication, with a size of 10.26 kb, in the *TBL1X *gene region. However, the lack of CNV identification in the *TBL1X *region might be due to the limitation of CNV identification algorithms based on GWAS data [35].

The association at *TBL1X *is even more intriguing, given that it is seen only in the male-only analysis. The *P *values of the female-specific tests for the three significant markers given in Table 2 were all greater than 0.1 in the individual, joint and meta-analyses. This suggests the possibility of a recessive effect at this locus; however, analysis of affected females with a recessive model did not improve the statistical significance. Alternatively, the lack of significance in females could be explained by skewed, allele-specific X-chromosome inactivation, which has been suggested previously in autism [36], or simply by low power in the female subset due to its relatively small sample size compared to males, as shown in Additional file 4.

The *TBL1X *gene has not been reported in other association studies of ASD. This might be due to the limited sample sizes, as we have shown that the odds ratios of the SNPs are modest. Wang *et al*. [9] also analyzed the X-chromosome markers for ASD using the AGRE and ACC data sets for replication analysis and meta-analysis. The markers in *TBL1X *did not pass the meta-analysis threshold of 1 × 10^-4 ^in their studies. We increased the sample size by including the HIHG/CHGR data set as well as the AGRE and ACC data sets in the meta- and joint analyses, which increased the power of our association studies.

We also found that two candidate genes, *DMD *and *IL1RAPL2*, were significantly associated with the discovery and validation data sets based on *a priori *hypotheses at previously identified candidate genes. The finding of a significant SNP (rs721699 in the *DMD *gene) warrants mention in light of recent findings in which exon duplications in the *DMD *gene were found to give rise to an autism phenotype [37]. The results reported by Pagnamenta *et al*. [38] support previous work by Hendriksen and Vles [39] in showing an increased rate, relative to the general population, of autism features in individuals with *DMD*. Although this finding is most likely a result of LD with functional variants in *DMD*, it adds to the growing body of work suggesting that autism may co-occur with other neurological conditions. Moreover, investigators in a recent study found a hemizygous deletion in the *DMD *gene in a male who had been diagnosed with ASD and later with muscle weakness [40]. Researchers in other studies have also suggested that *IL1RAPL1*, which is closely related to the *IL1RAPL2 *gene, identified in this study, is associated with autism [41,42].

Wang *et al*. [9] found that the three markers rs11798405, rs5972577 and rs6646569 on the X chromosome showedstatistically significant association with ASD, with *P *values less than 10^-5 ^based on meta-analysis of individual *P *values for AGRE and ACC. However, we observed significantly higher missing genotype rates for SNPs rs5972577 and rs6646569 in males than in females consistently across the HIHG/CHGR, AGRE and ACC data sets. As discussed in Additional file 8, these higher missing genotype rates can cause spurious association results. The problem of differential missingness between the sexes can be eliminated in sex-matched case-control studies. However, for family-based studies or case-control studies not matched on sex, the patterns of missing data between the sexes should be carefully examined, especially for sex-linked chromosomes. In Additional file 9, we show that the missing genotype rates for the markers in Table 2 are very low, which suggests that the statistical significance for these markers are not a result of the informative missingness. The proportion of markers (for example, 4.3% in the HIHG data set with *P *values less than 0.05 for the missingness tests between males and females) on the X chromosome that showed this differential missingness is what we expected by chance alone, which suggests that there is no systemic defect in the genotype calling algorithm for the X chromosome. The differential missingness between the sexes may be due to sequence homology or CNVs, which can introduce outliers into the intensity plots [43]. We set the threshold for missing genotypes at 2.5% for either males or females for each SNP in our QC procedure to minimize the effects of this problem. This resulted in the removal of SNPs rs5972577 and rs6646569 from our analyses. We also found that reclustering the samples without reference samples before genotype calling reduced the effect of the nonrandom and allele-specific missingness on association tests. For example, rs11798405 did not show significant results (*P *value greater than 0.05) in our reclustered AGRE samples, though it had a *P *value = 0.0067 in the study be Wang *et al*. [9]. In another ASD study [44], none of the markers on the X chromosome were reported to be significant. This could be due to the smaller sample size used in the study (2,394 probands) than we had in our study (3,503 affected individuals).

## Conclusion

This study has identified a gene associated with ASD, *TBL1X*. This gene showed modest but consistent effects in a family-based discovery data set and an independent case-control validation data set comprising males. Combined functional evidence with respect to pathways and prior evidence of this region involved with variable phenotypes suggests that further studies, such as fine-mapping of the gene, could identify ASD-associated regulatory variants, protein-altering rare variants and CNVs.

## Abbreviations

IL: interleukin; kb: kilobase; Mb: megabase; SNP: single-nucleotide polymorphism.

## Competing interests

The authors declare that they have no competing interests.

## Authors' contributions

All coauthors contributed to the writing of the manuscript. RHC was the primary author of the manuscript, developed the statistical methods used and conducted the analyses. DM, KW and JMJ contributed to the design of the analysis and the interpretation of the analysis results. DJH and JRG contributed to the molecular analysis and interpretation. MLC analyzed the clinical data and contributed to the study design. IK and PLW performed the molecular experiments. HHW, RKA, GDS, HH, JLH and MPV provided input regarding the study design and statistical analyses. ERM contributed to the design of the study, the development of its methods, the coordination of statistical and molecular analyses and the interpretation of data. All authors read and approved the final manuscript.

## Supplementary Material

Additional file 1 **Detailed statistics for subphenotypes of the data sets**. Additional file 1 contains detailed statistics for the subphenotype information for the John P Hussman Institute for Human Genomics/Center for Human Genetics Research (HIHG/CHGR) and Autism Genetic Resource Exchange (AGRE) data sets. Click here for file

Additional file 2 **A list of candidate genes for ASD used in the candidate gene analysis**. Additional file 2 is a list of the 21 candidate genes for autism spectrum disorder used to calculate statistical significance in candidate genes on the X chromosome. Click here for file

Additional file 3 **Procedures for the statistical analyses**. Additional file 3 describes the procedures used in the three analyses (that is, joint analysis, meta-analysis and replication analysis). Click here for file

Additional file 4 **Quality control steps**. Additional file 4 lists the detailed statistics for the samples passing the quality control (QC) steps. Click here for file

Additional file 5 **LD pattern among SNPs in the *TBL1X *gene based on unrelated individuals**. Additional file 4 gives the linkage disequilibrium (LD) measures (*r^2^*) for the significant SNPs and surrounding SNPs in the transducin β-like 1X-linked (*TBL1X*) gene. Click here for file

Additional file 6 **Allele and genotype frequencies of parents, cases and controls for significant SNPs**. Additional file 6 shows the allele and genotype frequencies of parents, cases and controls for the significant SNPs reported in Tables 2 and 3. Click here for file

Additional file 7 **Power study results**. Additional file 7 shows the power curves under different relative risks, minor allele frequencies and disease models, given the sample sizes in our study. Click here for file

Additional file 8 **Differences in missing data between males and females in the study**. Additional file 8 describes the problem of differences in missing genotype data, with statistics and figures showing the problem. Click here for file

Additional file 9 **Missing genotype rates for the markers rs5934665, rs17321050 and rs2188766**. Additional file 9 lists the missing genotype rates for males, females and overall samples for the significant markers in *TBL1X*. Click here for file
