# Supplementary material for: An X chromosome-wide association study in autism families identifies TBL1X as a novel autism spectrum disorder candidate gene in males
Source: Mol Autism. 2011 Nov 4;2:18. doi: 10.1186/2040-2392-2-18 (PMC3305893; doi:10.1186/2040-2392-2-18)
Supplement: Additional file 1 — Detailed statistics for subphenotypes of the data sets. Additional file 1 contains detailed statistics for the subphenotype information for the John P Hussman Institute for Human Genomics/Center for Human Genetics Research (HIHG/CHGR) and Autism Genetic Resource Exchange (AGRE) data sets. [file 2040-2392-2-18-S1.DOC]

**Additional file 1. Detailed statistics for sub-phenotypes in the datasets**

|  | HIHG/CHGR |
| --- | --- |
| ASD | 768 |
| Autism | 602 |
| Aspergers | 159 |
| PDD-NOS | 7 |
| Mean IQ | 66.62 (18.12)^1^ |
| Affected Male/Female ratio | 4.97 |
| % verbal | 82.29% |

^1^Mean and standard deviation

|  | AGRE |
| --- | --- |
| ASD | 1424 |
| Autism | 1243 |
| Broad Spectrum | 125 |
| Not quite autism | 56 |
| Mean IQ | 55.27 (20.34) |
| Affected Male/Female ratio | 3.82 |
| % verbal | 65.72% |

Statistics for the sub-phenotype information for the ACC dataset can be found in the Supplementary information in Wang et al. (Nature 2009, 459: 528-533)
