# Supplementary material for: An X chromosome-wide association study in autism families identifies TBL1X as a novel autism spectrum disorder candidate gene in males
Source: Mol Autism. 2011 Nov 4;2:18. doi: 10.1186/2040-2392-2-18 (PMC3305893; doi:10.1186/2040-2392-2-18)
Supplement: Additional file 2 — A list of candidate genes for ASD used in the candidate gene analysis. Additional file 2 is a list of the 21 candidate genes for autism spectrum disorder used to calculate statistical significance in candidate genes on the X chromosome. [file 2040-2392-2-18-S2.DOC]

**Additional file 2: a list of candidate genes for ASD used in the candidate gene analysis**

| AP1S2 |
| --- |
| ARX |
| ASMT |
| CDKL5 |
| DMD |
| FMR1 |
| GRPR |
| HOPA |
| IL1RAPL1 |
| IRAK1 |
| JARID1C |
| MAOA |
| MECP2 |
| NLGN3 |
| NLGN4 |
| PTCHD1 |
| RPL10 |
| SLC6A8 |
| SLC9A6 |
| STS |
| UPF3B |
|  |
