# Supplementary material for: An X chromosome-wide association study in autism families identifies TBL1X as a novel autism spectrum disorder candidate gene in males
Source: Mol Autism. 2011 Nov 4;2:18. doi: 10.1186/2040-2392-2-18 (PMC3305893; doi:10.1186/2040-2392-2-18)
Supplement: Additional file 3 — Procedures for the statistical analyses. Additional file 3 describes the procedures used in the three analyses (that is, joint analysis, meta-analysis and replication analysis). [file 2040-2392-2-18-S3.PPT]

## Slide 1
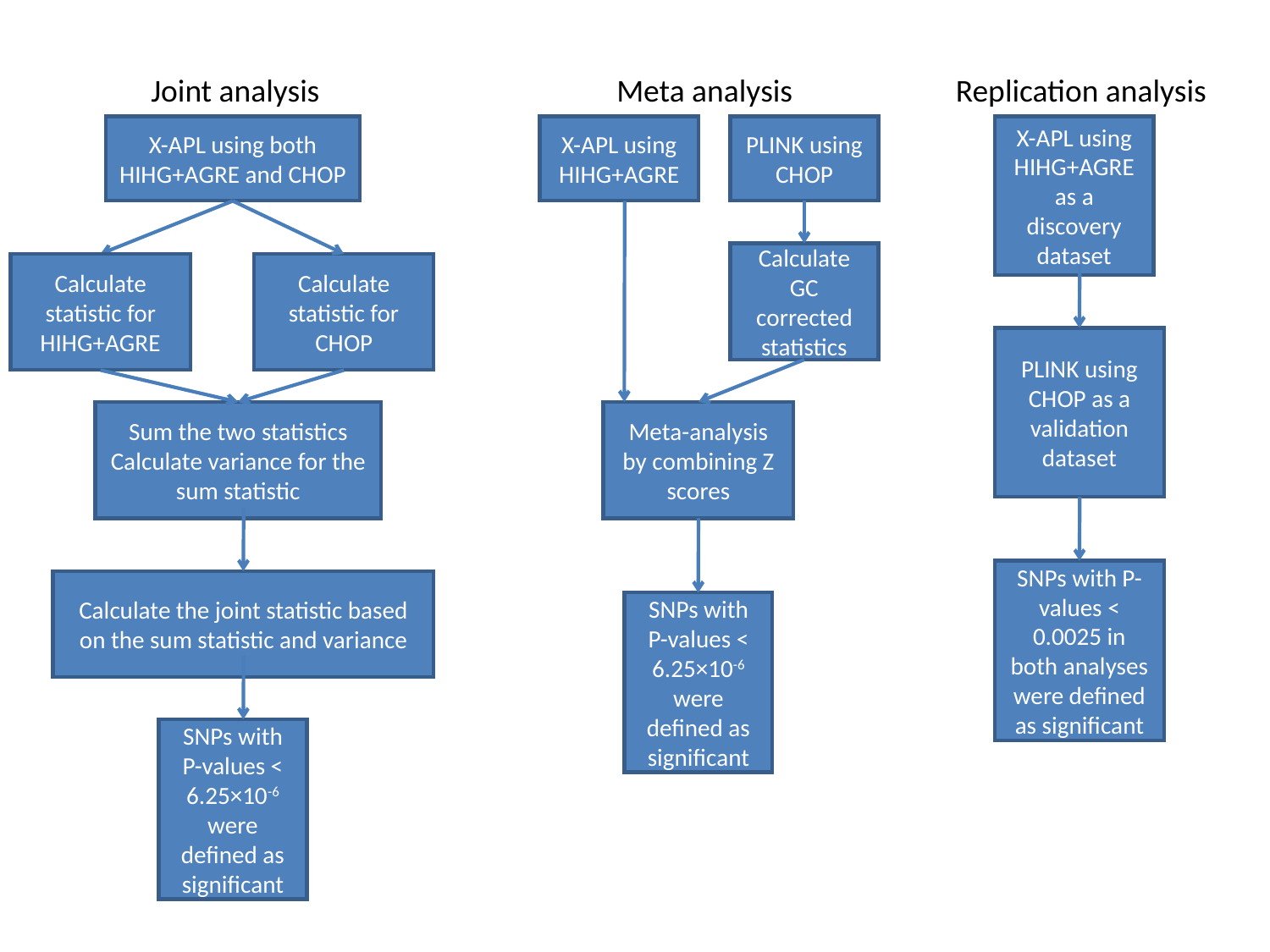

Joint analysis
Meta analysis
Replication analysis
X-APL using both HIHG+AGRE and CHOP
X-APL using HIHG+AGRE
PLINK using CHOP
X-APL using HIHG+AGRE as a discovery dataset
Calculate GC corrected statistics
Calculate statistic for HIHG+AGRE
Calculate statistic for CHOP
PLINK using CHOP as a validation dataset
Sum the two statistics
Calculate variance for the sum statistic
Meta-analysis by combining Z scores
SNPs with P-values < 0.0025 in both analyses were defined as significant
Calculate the joint statistic based on the sum statistic and variance
SNPs with P-values < 6.25×10^-6^ were defined as significant
SNPs with P-values < 6.25×10^-6^ were defined as significant
