# Supplementary material for: An X chromosome-wide association study in autism families identifies TBL1X as a novel autism spectrum disorder candidate gene in males
Source: Mol Autism. 2011 Nov 4;2:18. doi: 10.1186/2040-2392-2-18 (PMC3305893; doi:10.1186/2040-2392-2-18)
Supplement: Additional file 4 — Quality control steps. Additional file 4 lists the detailed statistics for the samples passing the quality control (QC) steps. [file 2040-2392-2-18-S4.DOC]

**Additional file 4**. Quality Control (QC) steps.

| SAMPLE QC | HIHG/CHGR | AGRE | ACC |
| --- | --- | --- | --- |
| Sample genotyped | 3,128 | 4,495 | 8,530 |
| Nuclear families genotyped | 894 | 939 |  |
| Sample dropped for QC | | | |
| Sample with low call rate (<95%) | 55 | 58 | 26 |
| Families with more than 2% Mendelian errors | 7 | 6 |  |
| Samples excluded based on race or substructure | 466 | 960 |  |
| Individuals tested for association | 2,557 | 3,289 | 7,676 |
| Families tested for association | 735 | 721 |  |
| Affected males (siblings/cases) | 726 | 1,131 | 989 |
| Affected females (siblings/cases) | 146 | 296 | 215 |
| Unaffected males (siblings or parents/controls) | 843 | 878 | 3,393 |
| Unaffected females (siblings or parents/controls) | 842 | 984 | 3,079 |
| GENOTYPE QC | | | |
| Successfully genotyped SNPs on the X chromosome | 36377 | 13287 | 11977 |
| SNPs with MAF < 0.01 | 11,400 | 273 | NA |
| SNPs with HWE p-value<10^-4^ | 15 | 401 | NA |
| SNPs with > 1% male heterozygotes^1^ | 63 | 4 | 20 |
| SNPs with missing rate > 2.5% in either males or females | 343 | 1,445 | 859 |
| SNPs tested for association | 24,712 | 11,164 | 11,098 |

^1^SNPs with > 1% male heterozygotes outside the PAR1 and PAR2 regions
