# Supplementary figures and images for: An X chromosome-wide association study in autism families identifies TBL1X as a novel autism spectrum disorder candidate gene in males
Source: Mol Autism. 2011 Nov 4;2:18. doi: 10.1186/2040-2392-2-18 (PMC3305893; doi:10.1186/2040-2392-2-18)

**Additional file 5. LD pattern among SNPs in the TBL1X gene based on unrelated individuals.**


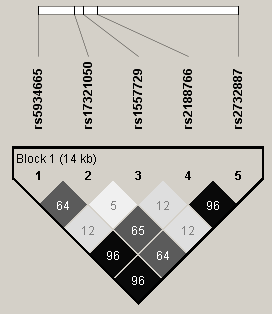

Supplement: Additional file 5 — LD pattern among SNPs in the TBL1X gene based on unrelated individuals. Additional file 4 gives the linkage disequilibrium (LD) measures (r2) for the significant SNPs and surrounding SNPs in the transducin β-like 1X-linked (TBL1X) gene. [file 2040-2392-2-18-S5.DOC]
