# Supplementary material for: An X chromosome-wide association study in autism families identifies TBL1X as a novel autism spectrum disorder candidate gene in males
Source: Mol Autism. 2011 Nov 4;2:18. doi: 10.1186/2040-2392-2-18 (PMC3305893; doi:10.1186/2040-2392-2-18)
Supplement: Additional file 6 — Allele and genotype frequencies of parents, cases and controls for significant SNPs. Additional file 6 shows the allele and genotype frequencies of parents, cases and controls for the significant SNPs reported in Tables 2 and 3. [file 2040-2392-2-18-S6.DOC]

**Additional file 6. Allele and Genotype frequencies for parents, cases and controls for the significant SNPs**

|  | rs5934665 | rs17321050 | rs2188766 | rs721699 | rs9887672 | rs10218388 | rs5962575 |
| --- | --- | --- | --- | --- | --- | --- | --- |
| Allele freq in parents (HIHG+AGRE) | 0.438 | 0.341 | 0.438 | 0.385 | 0.106 | 0.107 | 0.103 |
| Allele freq in affected siblings  (HIHG+AGRE) | 0.423 | 0.326 | 0.420 | 0.390 | 0.119 | 0.119 | 0.117 |
| Allele freq in male affected siblings  (HIHG+AGRE) | 0.400 | 0.297 | 0.397 | 0.391 | 0.128 | 0.128 | 0.125 |
| Allele freq in female affected siblings  (HIHG+AGRE) | 0.480 | 0.397 | 0.478 | 0.388 | 0.099 | 0.099 | 0.097 |
| Allele freq in controls (ACC) | 0.467 | 0.368 | 0.468 | 0.356 | 0.101 | 0.101 | 0.096 |
| Allele freq in cases (ACC) | 0.422 | 0.318 | 0.418 | 0.385 | 0.127 | 0.127 | 0.121 |
| Allele freq in male cases (ACC) | 0.414 | 0.303 | 0.408 | 0.376 | 0.132 | 0.132 | 0.128 |
| Allele freq in female cases  (ACC) | 0.438 | 0.351 | 0.442 | 0.407 | 0.116 | 0.115 | 0.103 |
| Genotype freq in parents  (HIHG+AGRE) | 0.171/  0.503/  0.326 | 0.102/  0.447/  0.451 | 0.171/  0.504/  0.325 | 0.153/  0.454/  0.393 | 0.017/  0.198/  0.785 | 0.017/  0.198/  0.785 | 0.013/  0.196/  0.791 |
| Genotype freq in female affected siblings  (HIHG+AGRE) | 0.218/  0.522/  0.260 | 0.133/  0.526/  0.341 | 0.215/  0.526/  0.259 | 0.138/  0.500/  0.362 | 0.016/  0.166/  0.818 | 0.016/  0.166/  0.818 | 0.016/  0.162/  0.822 |
| Genotype freq in female controls  (ACC) | 0.221/  0.482/  0.297 | 0.134/  0.464/  0.402 | 0.220/  0.486/  0.294 | 0.128/  0.466/  0.406 | 0.009/  0.189/  0.802 | 0.009/  0.187/  0.804 | 0.010/  0.178/  0.812 |
| Genotype freq in female cases  (ACC) | 0.200/  0.474/  0.326 | 0.130/  0.442/  0.428 | 0.196/  0.491/  0.313 | 0.149/  0.516/  0.335 | 0.009/  0.214/  0.777 | 0.009/  0.210/  0.781 | 0.009/  0.187/  0.804 |
