# Supplementary material for: An X chromosome-wide association study in autism families identifies TBL1X as a novel autism spectrum disorder candidate gene in males
Source: Mol Autism. 2011 Nov 4;2:18. doi: 10.1186/2040-2392-2-18 (PMC3305893; doi:10.1186/2040-2392-2-18)
Supplement: Additional file 7 — Power study results. Additional file 7 shows the power curves under different relative risks, minor allele frequencies and disease models, given the sample sizes in our study. [file 2040-2392-2-18-S7.DOC]

**Additional file 7. Power study results.**


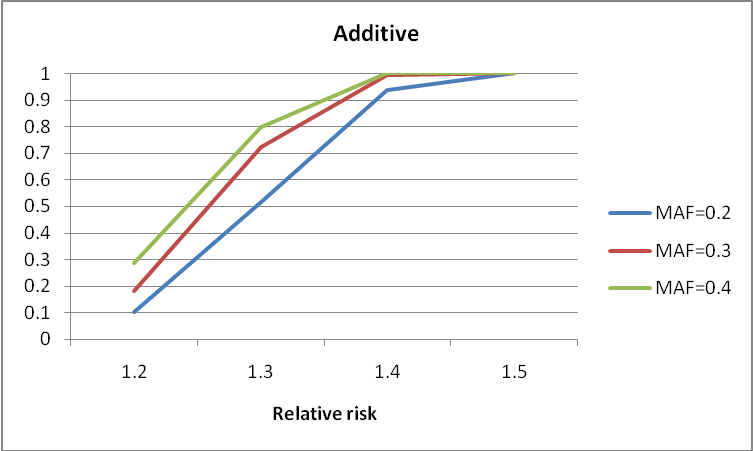


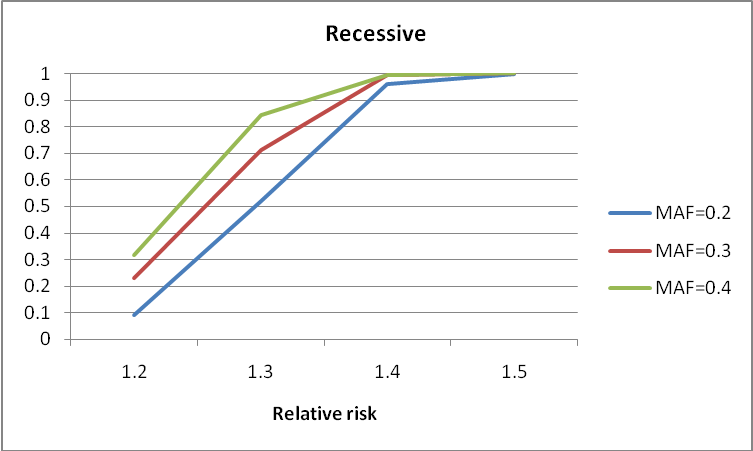


We simulated the same numbers of families in the HIHG/CHGR and AGRE datasets and the same numbers of cases and controls in the ACC dataset. The disease prevalence was specified as 0.008 for males and 0.002 for females. The disease locus only had effects on males. We simulated disease allele frequencies between 0.2-0.4 and disease relative risk between 1.2-1.5. Both additive and recessive models were simulated. The datasets were simulated with the simulation software SIMLA. A total of 500 replicates of simulated datasets were used to calculate the power for each scenario.
