# Supplementary material for: An X chromosome-wide association study in autism families identifies TBL1X as a novel autism spectrum disorder candidate gene in males
Source: Mol Autism. 2011 Nov 4;2:18. doi: 10.1186/2040-2392-2-18 (PMC3305893; doi:10.1186/2040-2392-2-18)

**Additional file 8: Differential missingness between males and females**

Wang et al. (Wang,K. 2009) showed that the three markers, rs11798405, rs5972577 and rs6646569, on the X chromosome were significant with p-values < 10^-5^ based on the meta-analysis of individual p-values for AGRE and ACC. However, we observed that the SNPs rs5972577 and rs6646569 have significantly higher missing rates in males than females consistently across the HIHG/CHGR, AGRE and ACC datasets as shown in the following table. We examined the genotype intensity plots from Illumina GenomeStudio and observed that the missingness was associated with a specific genotype for the three SNPs as shown in the following figure. We manually reclustered and recalled the genotypes for the three SNPs and confirmed that a significantly higher proportion of the recalled genotypes carry the minor alleles compared to the expected proportion in males based on the allele frequency (data not shown). We also sequenced 100 randomly selected individuals with missing genotypes for the three SNPs and found that the sequenced genotypes were all concordant with the manually recalled genotypes. Since males have higher prevalence of ASD than females, as seen in our datasets, a non-random missingness between males and females and the missingness specific to an allele can cause difference in allele frequencies between affecteds and unaffecteds. We performed association tests on the manually recalled genotypes for the three markers based on the same samples used in Wang et al. (Wang,K. 2009) and only rs11798405 showed a marginally significant p-value 0.03 in the AGRE dataset, as opposed to the p-value 0.0067 for the marker shown in Wang et al (Wang,K. 2009). This further verified that the non-random missingness and missingness specific to an allele can cause spurious association results.

Genotype missing rates for two SNPs reported in Wang et al. on the X chromosome

| Datasets |  | rs5972577 | rs6646569 |
| --- | --- | --- | --- |
| HIHG/CHGR | F_MALE^1^ | 0.011 | 0.021 |
|  | F_FEMALE^2^ | 0 | 0.002 |
|  | P-value^3^ | 0.03 | 0.006 |
| AGRE | F_MALE | 0.095 | NA^4^ |
|  | F_FEMALE | 0.086 | NA |
|  | P-value | 0.87 | NA |
| ACC | F_MALE | 0.077 | 0.066 |
|  | F_FEMALE | 0.005 | 0.018 |
|  | P-value | 1.26×10^-39^ | 3.34×10^-21^ |

^1^Missing rate in males

^2^Missing rate in females

^3^Missingness test p-value between unrelated males and females

^4^SNP was removed due to low quality score

Intensity plots for the three significant markers reported in Wang et al. (Wang,K. 2009)


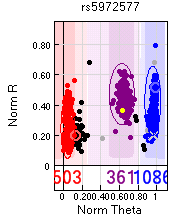

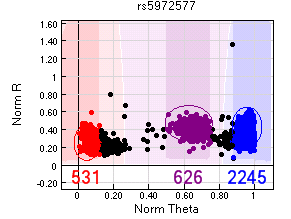


(CAP)

(AGRE)

(CAP)

(AGRE)


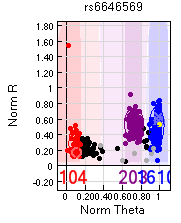

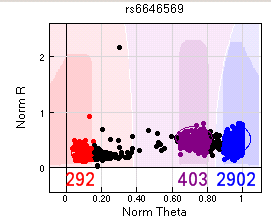


(CAP)

(AGRE)


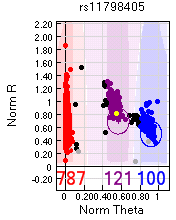

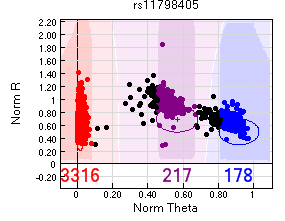

Supplement: Additional file 8 — Differences in missing data between males and females in the study. Additional file 8 describes the problem of differences in missing genotype data, with statistics and figures showing the problem. [file 2040-2392-2-18-S8.DOC]
