# Supplementary material for: An X chromosome-wide association study in autism families identifies TBL1X as a novel autism spectrum disorder candidate gene in males
Source: Mol Autism. 2011 Nov 4;2:18. doi: 10.1186/2040-2392-2-18 (PMC3305893; doi:10.1186/2040-2392-2-18)
Supplement: Additional file 9 — Missing genotype rates for the markers rs5934665, rs17321050 and rs2188766. Additional file 9 lists the missing genotype rates for males, females and overall samples for the significant markers in TBL1X. [file 2040-2392-2-18-S9.DOC]

**Additional file 9: missing rates for the markers rs5934665, rs17321050, and rs2188766**

The following table shows the missing rates for the three markers in TBL1X reported in Table 2.

|  | Discovery dataset | | | Validation dataset | | |
| --- | --- | --- | --- | --- | --- | --- |
|  | Males | Females | Overall | Males | Females | Overall |
| rs5934665 | 0.0031 | 0.0035 | 0.0033 | 0.0098 | 0.0109 | 0.0105 |
| rs17321050 | 0.0016 | 0.0009 | 0.0014 | 0.0000 | 0.0000 | 0.0000 |
| rs2188766 | 0.0014 | 0.0018 | 0.0015 | 0.0006 | 0.0006 | 0.0006 |

The table shows that the missing rates for the three markers are very low, and the missing rates between males and females are similar. Therefore, the missingness should have very limited effects on the validity of the tests. In order to further increase the power and eliminate any possible effects of informative missingness, we imputed the missing genotypes using the software IMPUTE v1. The HapMap CEU samples were used as the reference samples. Family members were treated as independent in the imputation. Imputed genotypes with the highest probabilities > 0.9 were called. Mendelian errors were checked for the imputed genotypes with PLINK for the family data. No Mendelian errors were found for the imputed genotypes. The following table shows the missing rates and p-values of the association tests for the three markers after the imputation.

|  | Discovery dataset | | | Validation dataset | | | Meta | Joint |
| --- | --- | --- | --- | --- | --- | --- | --- | --- |
|  | Males | Females | Overall | Males | Females | Overall |  |  |
| rs5934665 | 0.0011 | 0.0008 | 0.0010 | 0.0004 | 0.0000 | 0.0002 | 1.34×10^-5^ | 2.27×10^-5^ |
| rs17321050 | 0.0014 | 0.0008 | 0.0011 | 0.0000 | 0.0000 | 0.0000 | 4.32×10^-6^ | 1.27×10^-5^ |
| rs2188766 | 0.0011 | 0.0017 | 0.0013 | 0.0000 | 0.0000 | 0.0000 | 1.09×10^-5^ | 2.13×10^-5^ |

After the imputation, the missing rates between males and females are also very close and are close to 0. The p-values for the meta- and joint analyses are similar to the p-values before the imputation. This again demonstrates that the missingness does not affect the validity of the tests.
